# Immunobiotic Lactobacilli Improve Resistance of Respiratory Epithelial Cells to SARS-CoV-2 Infection

**DOI:** 10.3390/pathogens10091197

**Published:** 2021-09-15

**Authors:** Md. Aminul Islam, Leonardo Albarracin, Mikado Tomokiyo, Juan Carlos Valdez, Jacinto Sacur, Maria Guadalupe Vizoso-Pinto, Bruno G. N. Andrade, Rafael R. C. Cuadrat, Haruki Kitazawa, Julio Villena

**Affiliations:** 1 Food and Feed Immunology Group, Laboratory of Animal Food Function, Graduate School of Agricultural Science, Tohoku University, Sendai 981-8555, Japan; aminul.vmed@bau.edu.bd (M.A.I.); mikado0403@gmail.com (M.T.); 2 Department of Medicine, Faculty of Veterinary Science, Bangladesh Agricultural University, Mymensingh 2202, Bangladesh; 3 Laboratory of Immunobiotechnology, Reference Centre for Lactobacilli (CERELA-CONICET), Tucuman 4000, Argentina; lalbarracin@herrera.unt.edu.ar; 4 Laboratory of Immunology, Faculty of Biochemistry, Chemistry and Pharmacy, National University of Tucuman, Tucuman 4000, Argentina; pumavaldez@hotmail.com; 5 Infection Biology Laboratory, Instituto Superior de Investigaciones Biológicas (INSIBIO), CONICET-UNT, Tucuman 4000, Argentina; jacintosacur@gmail.com (J.S.); mgvizoso@fm.unt.edu.ar (M.G.V.-P.); 6 AdaptCentre, Munster Technological University (MTU), T12 P928 Cork, Ireland; Bruno.Andrade@cit.ie; 7 Max-Delbrück-Center for Molecular Medicine in the Helmholtz Association (MDC), Berlin Institute for Medical Systems Biology (BIMSB), 13125 Berlin, Germany; rafaelcuadrat@gmail.com; 8 Department of Molecular Epidemiology, German Institute of Human Nutrition Potsdam-Rehbrücke, 14558 Nuthetal, Germany; 9 Livestock Immunology Unit, International Education and Research Center for Food and Agricultural Immunology (CFAI), Graduate School of Agricultural Science, Tohoku University, Sendai 981-8555, Japan

**Keywords:** *Lactiplantibacillus plantarum*, respiratory epithelial cells, probiotics, immunobiotics, SARS-CoV-2, COVID-19, coronavirus, TLR3

## Abstract

Previously, we reported that immunomodulatory lactobacilli, nasally administered, beneficially regulated the lung antiviral innate immune response induced by Toll-like receptor 3 (TLR3) activation and improved protection against the respiratory pathogens, influenza virus and respiratory syncytial virus in mice. Here, we assessed the immunomodulatory effects of viable and non-viable *Lactiplantibacillus plantarum* strains in human respiratory epithelial cells (Calu-3 cells) and the capacity of these immunobiotic lactobacilli to reduce their susceptibility to the acute respiratory syndrome coronavirus 2 (SARS-CoV-2) infection. Immunobiotic *L. plantarum* MPL16 and CRL1506 differentially modulated IFN-β, IL-6, CXCL8, CCL5 and CXCL10 production and *IFNAR2*, *DDX58*, *Mx1* and *OAS1* expression in Calu-3 cells stimulated with the TLR3 agonist poly(I:C). Furthermore, the MPL16 and CRL1506 strains increased the resistance of Calu-3 cells to the challenge with SARS-CoV-2. *L. plantarum* MPL16 induced these beneficial effects more efficiently than the CRL1506 strain. Of note, neither non-viable MPL16 and CRL1506 strains nor the non-immunomodulatory strains *L. plantarum* CRL1905 and MPL18 could modify the resistance of Calu-3 cells to SARS-CoV-2 infection or the immune response to poly(I:C) challenge. To date, the potential beneficial effects of immunomodulatory probiotics on SARS-CoV-2 infection and COVID-19 outcome have been extrapolated from studies carried out in the context of other viral pathogens. To the best of our knowledge, this is the first demonstration of the ability of immunomodulatory lactobacilli to positively influence the replication of the new coronavirus. Further mechanistic studies and in vivo experiments in animal models of SARS-CoV-2 infection are necessary to identify specific strains of beneficial immunobiotic lactobacilli like *L. plantarum* MPL16 or CRL1506 for the prevention or treatment of the COVID-19.

## 1. Introduction

The genetically diverse *Orthocoronavirinae* family circulates in many species including avian and mammalian species. Three dangerous human coronaviruses (CoV) have emerged in the past 20 years: severe acute respiratory syndrome CoV (SARS-CoV) in 2002, Middle East respiratory syndrome CoV (MERS-CoV) in 2012, and SARS-CoV-2 in 2019, which is the causal agent of coronavirus disease 2019 (COVID-19) that has affected global health during 2020 and 2021 [1]. A significant proportion of SARS-CoV-2-infected persons are asymptomatic, while clinical symptoms of COVID-19 range from relatively mild common cold-like illness such as cough, fever, myalgia and fatigue, to severe dyspnea, chest pain, multifocal pneumonia, acute respiratory distress syndrome (ARDS), and multi-organ dysfunction syndrome [2]. Because of the efficient transmission of SARS-CoV-2 in immune naïve populations across the world, the virus has infected more than 210 million persons worldwide and caused the death of almost 4.5 million individuals between December 2019 and August 2021.

Biochemical, genetic and genomic studies have demonstrated that the spike protein of SARS-CoV-2 mediates the coronavirus’ entry into the host cell. The receptor-binding domain (RBD) of the S protein of SARS-CoV-2 mediates the recognition and attachment of the virus to the host cells through its interaction with the human angiotensin-converting enzyme 2 (ACE2). Additionally, SARS-CoV-2 entry requires the efficient priming of the S protein by the serine protease TMPRSS2 [3]. Hence, cell populations in human tissues with higher ACE2 and TMPRSS2 expressions are more vulnerable to SARS-CoV-2 infection. In this regard, it was shown that SARS-CoV-2 replicates efficiently in the human nasopharynx, bronchus [4] and lung [5] because of its high capacity to infect ACE2^+^TMPRSS2^+^ respiratory epithelial cells.

The replication of the SARS-CoV-2 in the respiratory epithelium does not go unnoticed, but rather, triggers a series of responses mediated by the immune system. The host’s innate antiviral immune response is initiated when viral molecular patterns are recognized by host cell pattern recognition receptors (PRR), including Toll-like receptors (TLR) and retinoic acid-inducible gene (RIG)-I-like receptors. This response initiates the activation of signaling pathways that ultimately results in the production of type I interferons (IFN) as well as inflammatory cytokines and chemokines, which together are essential for an effective antiviral response that eliminates the pathogen [6,7,8]. Type I IFNs trigger a signaling cascade via their receptor (IFNAR) in an autocrine or paracrine manner, which induces phosphorylation of signal transducers and activators of transcription 1 (STAT1) and STAT2. IFNs signaling triggers the expression of hundreds of antiviral proteins and chemokines, inducing an antiviral state in host cells. In fact, impaired responses of type I IFNs and antiviral factors have been associated with more severe COVID-19 cases [9,10,11]. On the other hand, virus infections trigger the production of inflammatory cytokines and chemokines in the respiratory tract including interleukin (IL)-6, chemokine (C-C motif) ligand 2 (CCL2), CCL5, CCL8, chemokine (C-X-C motif) ligand 8 (CXCL8), CXCL9, CXCL16, and CXCL2. While these inflammatory factors play a pivotal role in suppressing virus replication by recruiting and activating immune cells, the inadequate production of inflammatory factors might contribute to the viral pathogenesis [12]. Thus, the severity of COVID-19 depends not only on the SARS-CoV-2-induced cellular injury, but also on the host immune response [13].

In recent years, we and others have demonstrated that respiratory immunity can be modulated by the nasal administration of immunomodulatory lactobacilli (immunobiotics) [14,15,16,17,18,19]. In this regard, several studies in murine models have shown that priming of the nasal mucosa with immunobiotics enhanced the resistance against viral infections including those produced by the respiratory syncytial virus (RSV) [17,18,19], influenza virus (IFV) [20], and pneumonia virus of mice (PVM) [14,15,16]. In line with the results in animal models, a prospective observational study reported a higher nasopharyngeal abundance of *Lactobacillus* in healthy infants when compared with infants with RSV-acute respiratory infections [21]. Furthermore, a relative overabundance of *Lactobacillus* spp. was detected in the respiratory tract of healthy infants who had reduced risk of developing childhood wheezing illnesses associated to RSV infection [22]. Among the lactobacilli strains that possess these beneficial immunomodulatory properties, there is a group of strains belonging to the species *Lactiplantibacillus plantarum* (Basonym *Lactobacillus plantarum*), which have great potential to enhance antiviral immunity (reviewed in Villena et al. [8]). In our work, nasal administration of *L. plantarum* CRL1506 to mice prior to the challenge with the TLR3 agonist poly(I:C) or RSV differentially modulated the respiratory innate immune response, which improved viral clearance and reduced inflammatory-mediated lung damage [17]. Interestingly, we also demonstrated that nasal priming with heat-killed *L. plantarum* CRL1506 was capable of modulating the respiratory antiviral immune response, indicating that viability is not an essential factor for immunobiotic lactobacilli to exert their protective effect [17].

These previous studies prompted speculation that immunomodulatory lactobacilli in general, and *L. plantarum* in particular, would be able to beneficially modulate the respiratory innate immune response against SARS-CoV-2. Thus, the aim of this work was to assess whether viable or non-viable immunomodulatory *L. plantarum* strains were capable of differentially modulating cytokine production and antiviral factor expression in respiratory epithelial cells stimulated with poly(I:C) or challenged with SARS-CoV-2, and in this way, reduce viral replication.

## 2. Materials and Methods

### 2.1. Immunomodulatory Lactobacilli

*Lactiplantibacillus plantarum* CRL1506 and *L. plantarum* CRL1905 belong to the CERELA Culture Collection (CERELA-CONICET, Tucuman, Argentina). *L. plantarum* MPL16 and *L. plantarum* MPL18 belong to the Food and Feed Immunology Group Culture Collection (Tohoku University, Sendai, Japan). For the growth of lactobacilli strains, Man–Rogosa–Sharpe (MRS) broth was used at 37 °C. For the in vitro immunomodulatory assays, overnight cultures were harvested by centrifugation and washed three times with sterile phosphate-buffered saline (PBS). Lactobacilli were counted in a Petroff-Hausser counting chamber and resuspended in Dulbecco’s Modified Eagle medium (DMEM) until use. The MPL16 and the CRL1506 strains were selected because of their immunomodulatory functions, while the CRL681 and MPL18 strains were selected as negative controls [23,24]. Non-viable *L. plantarum* CRL1506 and MPL16 were obtained by tyndallization in a water bath at 80 °C for 30 min, and the lack of bacterial growth was confirmed using MRS agar plates [17,18,19].

### 2.2. Cell Cultures

The human lung epithelial cell line Calu-3 (HTB-55, ATCC, Manassas, VA, USA), is derived from a human pulmonary adenocarcinoma. Calu-3 cells were grown in DMEM supplemented with 20% fetal bovine serum (FBS) and 1% non-essential amino acid solution (Gibco, Grand Island, NY, USA). The FBS was reduced to 10% and 1% penicillin/streptomycin (Gibco) was added to the medium in antiviral assays. The African green monkey kidney epithelial cell line Vero 76 (ATCC CRL-1587) was grown in minimal essential medium (MEM) supplemented with 10% FBS. The FBS was reduced to 2% in antiviral assays. The cells were incubated at 37 °C in a humidified incubator in an atmosphere with 5% CO_2_ as described previously [25].

### 2.3. Cytokine Concentrations in Culture Supernatants

For the evaluation of the effect of lactobacilli in the innate immune response induced by the activation of TLR3, 1 mL of DMEM containing the different *L. plantarum* strains (5 × 10^7^ cells/mL) were added to Calu-3 cells monolayers, which were cultured in 6-well plates at a density of 2 × 10^6^ cells/well. Cells were further incubated for 24 h at 37 °C, 5% CO_2_. Lactobacilli were removed by washing with PBS and the Calu-3 cells were stimulated with 15 μg/mL of poly(I:C) (Sigma-Aldrich, San Luis, MO, USA), with a synthetic double-stranded RNA (dsRNA) and TLR3 agonist [25]. Before the challenge with poly(I:C) (basal levels) and 48 h after TLR3 activation, culture supernatants were collected for the evaluation of cytokines by enzyme-linked immunosorbent assay (ELISA) technique kits as described previously [25].

The concentrations of IFN-β (sensitivity: 0.7 pg/mL), IL-6 (sensitivity: 0.7 pg/mL), CCL5 (or RANTES) (sensitivity: 6.6 pg/mL), CXCL8 (or IL-8) (sensitivity: 7.5 pg/mL) and CXCL10 (or IP-10) (sensitivity: 4.46 pg/mL) were measured with commercial ELISA kits according to the recommendations of the manufacturer (R&D Systems, Minneapolis, MN, USA). 

### 2.4. SARS-CoV-2 Infection 

Working stocks of SARS-CoV-2 (clinical isolate hCoV-19/USA/VA/2020) were obtained in Vero 76 cells. Calu-3 2B4, a subpopulation of Calu-3 cells with high ACE2 expression, were used in SARS-CoV-2 infection experiments. These cells were obtained by sorting with an ACE2 antibody as described previously [25,26,27]. Calu-3 2B4 cells were stimulated with 1 mL of DMEM containing the different *L. plantarum* strains (5 × 10^7^ cells/mL) for 24 h at 37 °C, 5% CO_2_. PBS washing was used to remove lactobacilli and then Calu-3 2B4 cells were infected with SARS-CoV-2 (MOI 0.1) at 37 °C. After 30 min, the unbound virus was removed by PBS washing, and cells were incubated for 48 or 72 h at 37 °C, and 5% CO_2_. The replication of SARS-CoV-2 in Calu-3 2B4 cells was evaluated at hours 48 and 72 post-infection, considering a previous report that stated that the replication kinetics of this virus in these cells is slower than that observed in Vero 76 cells [28].

The plaque-forming units (PFU) of infectious SARS-CoV-2 were quantified by plaque titration on Vero 76 cells, as described elsewhere [29,30,31] with minor modifications. Vero 76 were seeded in 24-well plates, washed with PBS, incubated with serial dilutions of SARS-CoV-2-containing cell culture supernatants in duplicates, and overlaid with 1.2% Avicel in DMEM. The cells were fixed with 6% formalin after 72 h and visualized by staining with crystal violet [25].

The lactate dehydrogenase (LDH), which is a cytosolic enzyme released upon damage to the plasma membrane was measured in Calu-3 2B4 cells’ supernatants as an indicator of cellular toxicity. The LDH assay kit (Weiner Lab, Boston, MA, USA) was used to determine LDH levels in the supernatants of Calu-3 2B4 cells. The concentrations of IFN-β, IL-6, CCL5, CXCL8 and CXCL10 were measured by ELISA kits as described above.

All the experiments were conducted in Biosafety Level-3 (BSL-3) facilities according to the biosafety guidance related to COVID-19 [32,33].

### 2.5. Quantitative Expression Analysis by Two-Step Real-Time Quantitative PCR

Two-step real-time quantitative PCR (qPCR) was performed to characterize the expression of immune factors in Calu-3 cells. The total RNA isolation from each Calu-3 cell sample was performed with TRIzol reagent (Invitrogen, Waltham, MA, USA). The Quantitect RT kit (Qiagen, Tokyo, Japan) was used for the synthesis of cDNAs following the recommendations of the manufacturer. Real-time qPCR was carried out using a 7300 real-time PCR system (Applied Biosystems, Warrington, UK) and the Platinum SYBR green qPCR SuperMix uracil-DNA glycosylase with 6-carboxyl-X-rhodamine (ROX) (Invitrogen). The primers for *TMPRSS2* (F: CAAGTGCTCCAACTCTGGGAT, R: AACACACCGATTCTCGTCCTC), *ACE2* (F: ACAGTCCACACTTGCCCAAAT, R: GAGAGCACTGAAGACCCATT), *DDX58* (F: TGCGAATCAGATCCCAGTGTA, R: TGCCTGTAACTCTATACCCATGT), and *IFNAR2* (F: TCATGGTGTATATCAGCCTCGT, R: AGTTGGTACAATGGAGTGGT TTT) were used according to Sun et al. [34]. The primers for *2’5’-OAS1* (F: AGGAAAGGTG CTTCCGAGGTAG, R: GGACTGAGGAAGACAACCAGGT) and *Mx1* (F: TTCAGCACCTGATGGCCTATC, R: TGGATGATCAAAGGGATGTGG) were used according to Shuai et al. [35].

The PCR cycling conditions were 2 min at 50 °C, followed by 2 min at 95 °C, and then 40 cycles of 15 s at 95 °C, 30 s at 60 °C, and 30 s at 72 °C. The reaction mixtures contained 5 mL of sample cDNA and 15 mL of master mix, which included the sense and antisense primers. The expression of housekeeping gene *ATF4* (F: CTCCGGGACAGATTGGATGTT, R: GGCTGCTTATTAGTCTCCTGGAC) with no differential expression during SARS-CoV-2 infection in Calu-3 cells [34] was used as reference gene.

### 2.6. Statistical Analysis

Results were expressed as mean ± standard deviation (SD) from experiments made in triplicate. Normal distributed data were tested by 2-way ANOVA. Tukey’s test (for pairwise comparisons of the means) or the Fisher’s least significant difference (LSD) test (for multi-comparison) were used to evaluate the differences among groups. Differences were considered significant at *p* < 0.05.

## 3. Results

### 3.1. Immunobiotic L. plantarum Strains Differentialy Modulate the Response of Respiratory Epithelial Cells to Poly(I:C) Stimulation

**Figure 1:**
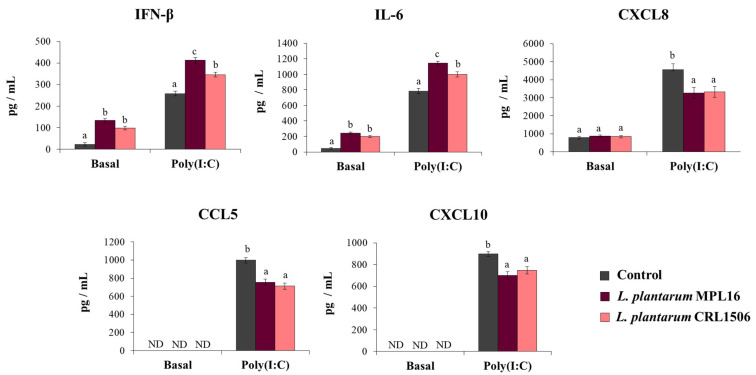
Influence of *L. plantarum* strains on the antiviral immune response of respiratory epithelial cells induced by Toll-like receptor 3 (TLR3) signaling activation. Calu-3 cells were treated with *L. plantarum* MPL16 or CRL1506 (5 × 10^7^ cells/mL) for 24 h and then stimulated with poly(I:C) for 48 h. Respiratory epithelial cells with no lactobacilli treatment were used as controls. Before (basal groups) and after (poly(I:C) groups) TLR3 activation, the culture supernatants were collected for cytokines´ determination using ELISA. ND: not detected. The results represent data from 3 independent experiments. Letters indicate significant differences (*p* < 0.05), a < b < c.

Our first aim was to study the effect of immunomodulatory *L. plantarum* strains on the response of respiratory epithelial cells to poly(I:C) challenge. The treatment of Calu-3 cells with *L. plantarum* MPL16 or CRL1506 did not cause detrimental effects or modify the culture supernatants’ LDH levels (data not shown). Before the stimulation with poly(I:C) (basal levels) the treatment of Calu-3 cells with both *L. plantarum* MPL16 and CRL1506 significantly increased IFN-β and IL-6 concentrations in the culture supernatants while no modifications in the levels of CXCL8 were observed (Figure 1). The MPL16 strain increased the basal production of IFN-β and IL-6 as efficiently as *L. plantarum* CRL1506. Basal levels of CXCL10 or CCL5 (Figure 1) were not detected in the supernatants of cultured Calu-3 cells by using the ELISA kits as described previously [25].

The effect of immunobiotic lactobacilli on the Calu-3 cells’ response to the stimulation with poly(I:C) was also evaluated. Challenging Calu-3 cells with poly(I:C) enhanced IFN-β levels as well as IL-6 and CXCL8 concentrations in control cells as we recently described [25]. Moreover, detectable concentrations of CXCL10 and CCL5 were found in the supernatants of poly(I:C)-challenged cells (Figure 1). The pre-treatment of respiratory epithelial cells with *L. plantarum* MPL16 or CRL1506 significantly enhanced IFN-β and IL-6 levels and reduced the concentration of the three chemokines when compared to control cells. Of note, the MPL16 strain was more efficient than *L. plantarum* CRL1506 in enhancing IFN-β and IL-6, while the two strains were equally effective in reducing CXCL8, CCL5 and CXCL10 (Figure 1).

### 3.2. Immunobiotic L. plantarum Strains Increase the Resistance of Respiratory Epithelial Cells to SARS-CoV-2 Infection

**Figure 2:**
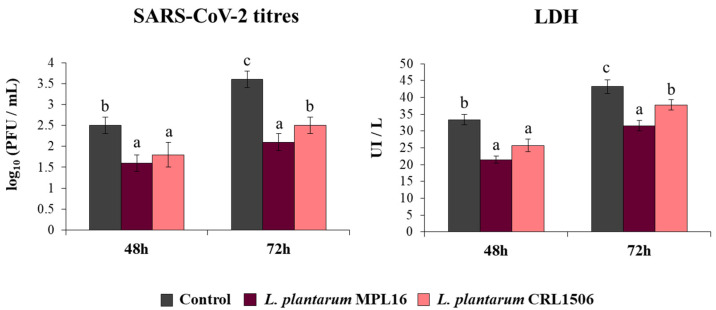
Influence of *L. plantarum* strains on the resistance of respiratory epithelial cells to infection with severe acute respiratory syndrome virus 2 (SARS-CoV-2). Calu-3 2B4 cells were treated with *L. plantarum* MPL16 or CRL1506 (5 × 10^7^ cells/mL) for 24 h and then infected with the coronavirus. Respiratory epithelial cells with no lactobacilli treatment were used as controls. SARS-CoV-2 titers and LDH levels in culture supernatants were evaluated at hours 48 and 72 post-infection. The results represent data from 3 independent experiments. Letters indicate significant differences (*p* < 0.05), a < b < c.

Coronaviruses are capable of synthetizing dsRNA molecules during their replication and mRNA transcription [36] and it was found that SARS-CoV-2 is detected by the antiviral systems that sense dsRNA in epithelial cells of the respiratory tract [28]. Furthermore, we recently reported that infection of Calu-3 2B4 cells with SARS-CoV-2 induces a profile of IFN-β and inflammatory cytokines and chemokines that resembles stimulation with poly(I:C) [25]. Then, considering the ability of immunobiotic lactobacilli to differentially modulate type I IFNs and cytokine profiles triggered by poly(I:C) stimulation in vivo [17,18,19] and in Calu-3 cells (Figure 1), we speculated that *L. plantarum* MPL16 and CRL1506 may affect SARS-CoV-2 replication in Calu-3 2B4 cells. Thus, we stimulated Calu-3 2B4 cells with viable *L. plantarum* MPL16 or CRL1506 and subsequently challenged the respiratory epithelial cells with SARS-CoV-2. Viral titers and LDH were evaluated at hours 48 and 72 post-infection (Figure 2). 

Replication of SARS-CoV-2 was observed at the two time points assessed, with higher titres at hour 72. Higher LDH levels were also found in the supernatants of infected cells at hour 72 when compared to hour 48. The pre-treatment of Calu-3 2B4 cells with immunobiotic lactobacilli significantly diminished the replication of the coronavirus as well as the values of LDH at hours 48 and 72 in comparison with the control group. Although both lactobacilli were equally effective in reducing coronavirus titres and LDH levels at hour 48 post-infection, *L. plantarum* MPL16 was more effective than the CRL1506 strain to reduce those parameters at hour 72 (Figure 2).

**Figure 3:**
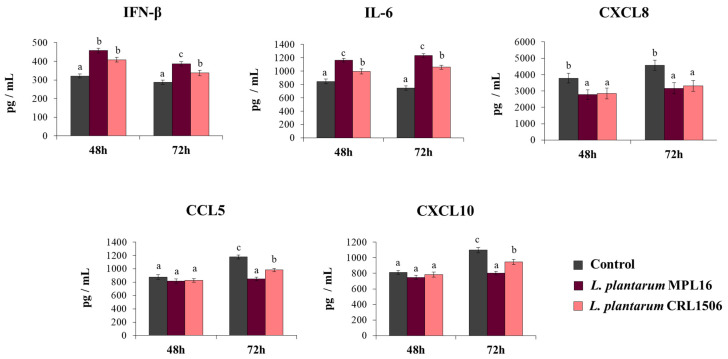
Influence of *L. plantarum* strains on the antiviral immune response of respiratory epithelial cells induced by the infection with severe acute respiratory syndrome virus 2 (SARS-CoV-2). Calu-3 2B4 cells were treated with *L. plantarum* MPL16 or CRL1506 (5 × 10^7^ cells/mL) for 24 h and then infected with the coronavirus. Respiratory epithelial cells with no lactobacilli treatment were used as controls. Culture supernatants were collected for cytokines´ determination using ELISA at hours 48 and 72 post-infection. The results represent data from 3 independent experiments. Letters indicate significant differences (*p* < 0.05), a < b < c.

To demonstrate that the improved resistance of immunobiotic-treated Calu-3 2B4 cells against the infection with SARS-CoV-2 was related to an enhanced antiviral immune response, we also determined IFN-β, IL-6, and chemokines concentrations in the culture supernatants of challenged cells (Figure 3). SARS-CoV-2 challenge induced the production of all the cytokines at the two-time points assessed. Of note, CXCL8, CCL5 and CXCL10 levels were greater at hour 48 than hour 72. The pre-treatment of Calu-3 2B4 cells with immunobiotic lactobacilli significantly enhanced IFN-β and IL-6 levels induced by the SARS-CoV-2 infection. While no differences in the levels of IFN-β and IL-6 were observed between MPL16- and CRL1506-treated cells at hour 48 post-infection, *L. plantarum* MPL16 was more efficient than the CRL1506 strain to increase the two immune factors at hour 72 post-infection (Figure 3). Both lactobacilli were equally effective in reducing CXCL8 concentrations at the two hours assessed, while no effect of lactobacilli treatments was detected when the levels of CCL5 and CXCL10 were analyzed at hour 48 post-infection (Figure 3). However, Calu-3 2B4 cells treated with MPL16 or CRL1506 strains produced significantly lower CCL5 and CXCL10 levels at hour 72 post-SARS-CoV-2 infection. Of note, *L. plantarum* MPL16 was more efficient than the CRL1506 strain in inducing this effect (Figure 3).

**Figure 4:**
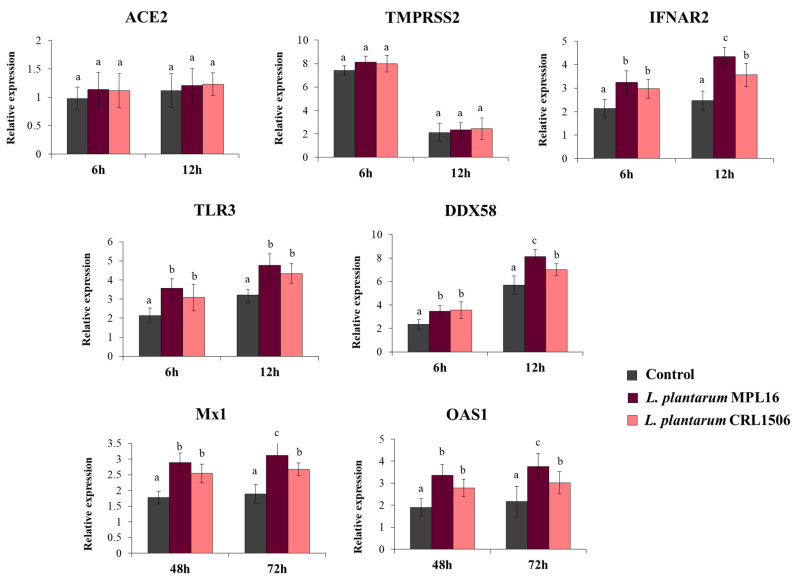
Influence of *L. plantarum* strains on the antiviral immune response of respiratory epithelial cells induced by the infection with severe acute respiratory syndrome virus 2 (SARS-CoV-2). Calu-3 cells were treated with *L. plantarum* MPL16 or CRL1506 (5 × 10^7^ cells/mL) for 24 h and then infected with the coronavirus. Respiratory epithelial cells with no lactobacilli treatment were used as controls. ACE2, TMPRSS2 and antiviral factors were determined by real-time qPCR at the indicated time points post-infection. ND: not detected. The results represent data from 3 independent experiments. Letters indicate significant differences (*p* < 0.05), a < b < c.

We also evaluated the expression of the receptors used by SARS-CoV-2 to invade respiratory epithelial cells at hours 6 and 12 post-infection, since it has been suggested that the coronavirus can modulate their expression earlier after the challenge of Calu-3 cells [34]. While the expression levels of *ACE2* were similar in the two time points assessed, the expression of *TMPRSS2* was significantly higher at hour 6 than hour 12 (Figure 4). No differences were found when *ACE2* and *TMPRSS2* expression in lactobacilli-treated cells were compared with controls. Increased expression of the PRRs *TLR3* and *DDX58,* as well as the receptor *IFNAR2* were found in Calu-3 cells infected with SARS-CoV-2, and the up-regulation was more notable at hour 12 than hour 6 (Figure 4). Both *L. plantarum* MPL16 and CRL1506 were able to significantly enhance the expression of *TLR3*, *DDX58* and *IFNAR2* when compared to control cells at the two-time points studied. However, the MPL16 strain was more efficient than *L. plantarum* CRL1506 in increasing *DDX58* and *IFNAR2* at hour 12 post-infection (Figure 4). In addition, Calu-3 cells increased their expression of *Mx1* and *OAS1* at hours 48 and 72 post-infection. MPL16 and CRL1506 treatments were able to significantly augment the expression of both antiviral factors when compared to control cells at the two time points assessed. However, *L. plantarum* MPL16 was more efficient than the CRL1506 strain in increasing *Mx1* and *OAS1* at hour 72 post-SARS-CoV-2 infection (Figure 4).

### 3.3. Live L. plantarum Strains Are Necessary for the Modulation of Antiviral Immune Response in Respiratory Epithelial Cells

**Figure 5:**
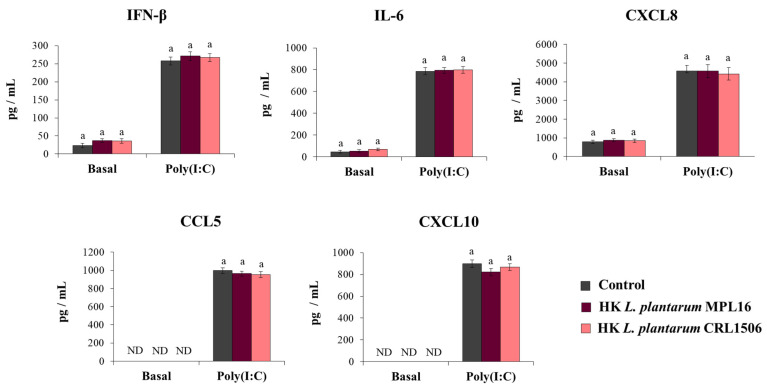
Influence of non-viable *L. plantarum* strains on the antiviral immune response of respiratory epithelial cells induced by Toll-like receptor 3 (TLR3) activation. Calu-3 cells were treated with heat-killed (HK) *L. plantarum* MPL16 or CRL1506 (5 × 10^7^ cells/mL) for 24 h and then stimulated with poly(I:C) for 48 h. Respiratory epithelial cells with no lactobacilli treatment were used as controls. Before (basal groups) and after (poly(I:C) groups) TLR3 activation, culture supernatants were collected for cytokines´ determination using ELISA. ND: not detected. The results represent data from 3 independent experiments. Similar letters indicate no significant differences (*p* < 0.05).

We reported previously that non-viable immunobiotic lactobacilli administered by the nasal route to mice were able to differentially modulate the respiratory innate antiviral immune response triggered by poly(I:C) administration or RSV infection [17,18,19]. Then, our next aim was to evaluate whether non-viable *L. plantarum* MPL16 or CRL1506 were able to modulate the response of Calu-3 cells to the activation of TLR3. For this purpose, respiratory epithelial cells were treated with heat-killed (HK) lactobacilli and then challenged with poly(I:C). Cytokines were evaluated in Calu-3 cells´ supernatants before and after TLR3 activation. As shown in Figure 5, the treatment of the respiratory epithelial cells with HK lactobacilli did not cause changes in the basal levels of IFN-β, IL-6, or the chemokines CXCL8, CCL5 and CXCL10. In addition, no differences in the concentrations of IFN-β, IL-6, or chemokines were found when control respiratory epithelial cells were compared with HK lactobacilli-treated cells after the stimulation with poly(I:C) (Figure 5).

**Figure 6:**
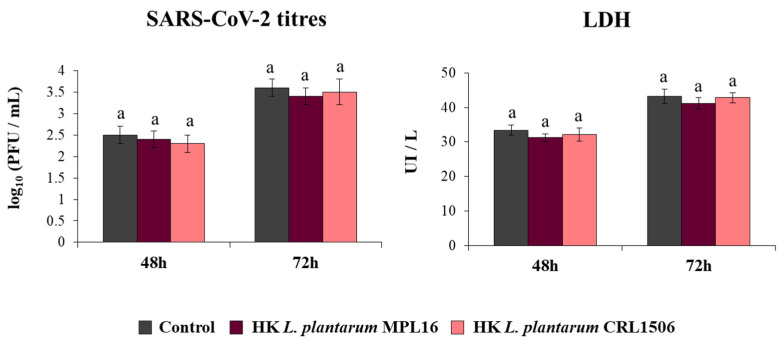
Influence of non-viable *L. plantarum* strains on the resistance of respiratory epithelial cells to the infection with severe acute respiratory syndrome virus 2 (SARS-CoV-2). Calu-3 2B4 cells were treated with heat-killed (HK) *L. plantarum* MPL16 or CRL1506 (5 × 10^7^ cells/mL) for 24 h and then infected with the coronavirus. Respiratory epithelial cells with no lactobacilli treatment were used as controls. SARS-CoV-2 titers and LDH levels in culture supernatants were determined at hours 48 and 72 post-infection. The results represent data from 3 independent experiments. Similar letters indicate no significant differences (*p* < 0.05).

We also aimed to demonstrate that HK lactobacilli were not capable of influencing the response of respiratory epithelial cells to the coronavirus infection because of the lack of immunomodulatory activity prior to the poly(I:C) stimulation. To test this hypothesis, Calu-3 2B4 cells were pre-treated with HK *L. plantarum* MPL16 or CRL1506 and then infected with the coronavirus. As expected, neither HK MPL16 nor HK CRL1506 were capable of reducing SARS-CoV-2 titers or LDH levels (Figure 6). 

### 3.4. The Ability of L. plantarum to Modulate Antiviral Immunity in Respiratory Epithelial Cells Is a Strain-Dependent Property

**Figure 7:**
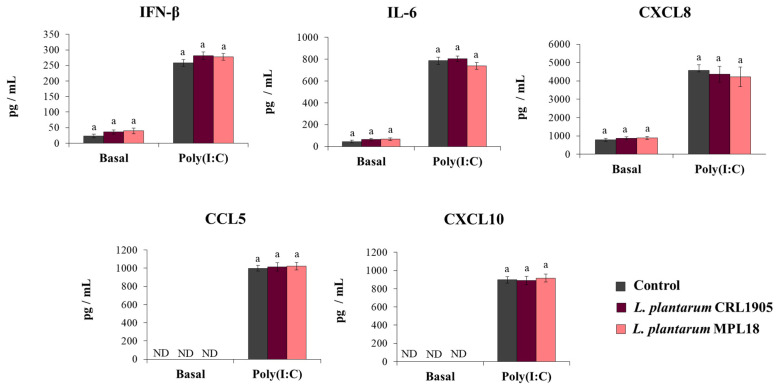
Influence of non-immunomodulatory *L. plantarum* strains on the antiviral immune response of respiratory epithelial cells induced by Toll-like receptor 3 (TLR3) activation. Calu-3 cells were treated with *L. plantarum* CRL1905 or MPL16 (5 × 10^7^ cells/mL) for 24 hours and then stimulated with poly(I:C) for 48 h. Respiratory epithelial cells with no lactobacilli treatment were used as controls. Before (basal groups) and after (poly(I:C) groups) TLR3 activation, culture supernatants were collected for cytokines´ determination using ELISA or antiviral factors using real-time qPCR. ND: not detected. The results represent data from 3 independent experiments. Similar letters indicate no significant differences (*p* < 0.05).

The effect of probiotic lactobacilli on the mucosal immune responses has been shown to be a strain-specific property. In fact, the immunomodulatory abilities of one Lactobacillus strain cannot be extrapolated to others, even of the same species [24,37]. Thus, we aimed to evaluate the effect of non-immunomodulatory L. plantarum strains on respiratory epithelial cells in the context of TLR3 activation. For this purpose, we stimulated respiratory epithelial cells with the non-immunomodulatory strains CRL1905 and MPL18 [23,24,38] and then challenged them with poly(I:C). As shown in Figure 7, L. plantarum CRL1905 and MPL18 were not able to modify the levels of IFN-β, IL-6, CXCL8, CCL5 or CXCL10 in the supernatants of Calu-3 cells before the challenge with poly(I:C). The CRL1905 and MPL18 strains were also unable to induce modifications in the concentrations of IFN-β, IL-6 and chemokines after the activation of TLR3 (Figure 7).

**Figure 8:**
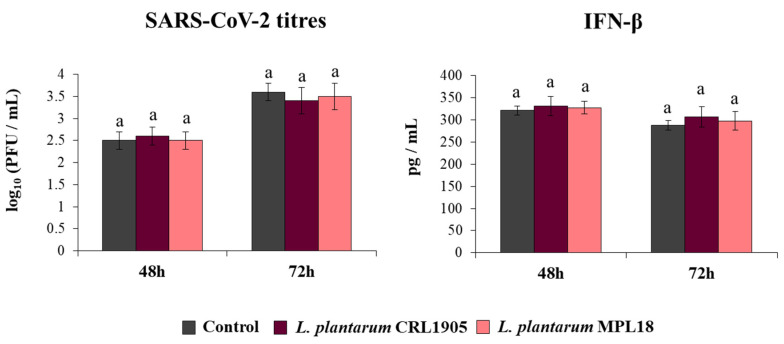
Influence of non-immunomodulatory *L. plantarum* strains on the resistance of respiratory epithelial cells to the infection with severe acute respiratory syndrome virus 2 (SARS-CoV-2). Calu-3 2B4 cells were treated with *L. plantarum* CRL1905 or MPL18 (5 × 10^7^ cells/mL) for 24 h and then infected with the coronavirus. Respiratory epithelial cells with no lactobacilli treatment were used as controls. SARS-CoV-2 titers and interferon (IFN)-β levels in culture supernatants were determined at hours 48 and 72 post-infection. The results represent data from 3 independent experiments. Similar letters indicate no significant differences (*p* < 0.05).

Finally, we assessed the ability of *L. plantarum* CRL1905 and MPL18 to modify the resistance of Calu-3 2B4 cells to the coronavirus challenge (Figure 8). Neither the CRL1905 nor the MPL18 strains were able to reduce coronavirus titres in infected respiratory epithelial cells at the two time points assessed. In line with these results, the levels of IFN-β in CRL1905- and MPL18-treated Calu-3 2B4 cells were not different compared to control cells at both hours 48 and 72 post-SARS-CoV-2 infection (Figure 8).

## 4. Discussion

As described for several respiratory viruses like IFV and RSV, the immune response plays a critical role in the outcome of SARS-CoV-2-induced pneumonia. Acute infection can induce an exacerbated disease due to immune-mediated pulmonary injury, resulting in severe morbidity and mortality in critically ill COVID-19 patients [12,13]. Therefore, identifying novel approaches to modulate virus-induced immunopathology would be beneficial in preventing or treating acute and lethal respiratory viral infections such as those produced by SARS-CoV-2. In this regard, our earlier studies revealed that nasally administered immunobiotic lactobacilli have the potential to beneficially influence the respiratory immune response in the context of viral infections [17,18,19]. In our hands, nasally administered *L. plantarum* CRL1506 was able to increase the levels of IFN-α, IFN-β, IL-6, and IFN-γ in the broncho-alveolar lavages of mice after the nasal challenge with poly(I:C) or the infection with RSV. These effects were associated with a significant reduction in viral replication and lung damage [17]. Furthermore, we demonstrated that the CRL1506 strain was able to increase MHC-II^+^CD11c^+^CD11b^low^CD103^+^ and MHC-II^+^CD11c^+^CD11b^high^CD103^-^ dendritic cells and CD3^+^CD4^+^IFN-γ^+^ T cells in the respiratory tract, indicating its ability to modulate the number and activity of immune cells. The experiments shown in this work represent a direct extension of our previous studies, which addressed the critical contributions of respiratory epithelial cells in the immunomodulatory activity of *L. plantarum* CRL1506 [17]. We observed here that the pretreatment of Calu-3 cells with immunobiotic *L. plantarum* strains significantly increased the production of IFN-β and IL-6 while it reduced CCL5, CXCL8 and CXCL-10. Thus, it is tempting to speculate that *L. plantarum* CRL1506 would be capable of interacting with both respiratory immune cells and epithelial cells when nasally administered; thus, inducing changes that improve antiviral defenses.

We also demonstrated here that the pretreatment of Calu-3 cells with immunobiotic *L. plantarum* strains increases their resistance to SARS-CoV-2 infection. To the best of our knowledge, no study has reported the effect of immunomodulatory probiotic lactobacilli against SARS-CoV-2 infection. One report used Vero E6 cells stimulated with lactobacilli for 2 h before the challenge with SARS-CoV-2 and evaluated the viability of infected cells by using the MTT test after 3 days post-infection [39]. The work described that the treatment of Vero E6 cells with *Limosilactobacillus fermentum* 90TC4 significantly increased the viability of cells challenged with SARS-CoV-2 when compared to infected controls. However, that work did not provide possible mechanism(s) to explain the beneficial effect of lactobacilli. Thus, we were interested in performing a more accurate evaluation of the potential protective effect of lactobacilli in the context of SARS-CoV-2 infection by using respiratory epithelial cells and evaluating the innate immune response. Here, we demonstrated that immunobiotic *L. plantarum* strains can influence the innate antiviral immune response of respiratory epithelial cells triggered by SARS-CoV-2 infection, improving the resistance of cells to the virus replication. 

High-throughput sequencing transcriptomic studies in Calu-3 cells revealed the rapid growth of SARS-CoV-2, which was accompanied by an early intensive response of the host genes [34]. It was found that SARS-CoV-2 did not induce changes in the expression of *ACE2* during the first 24 h of infection but significantly increased *TMPRSS2* in Calu-3 cells [34]. In line with those findings, we did not observe variations in the expression of *ACE2* in Calu-3 cells at hours 6 and 12 post-infection, while *TMPRSS2* was significantly up-regulated at hour 6. Since ACE2 and TMPRSS2 were reported to be essential for SARS-CoV-2 entry to susceptible cells [4], one possible mechanism involved in the ability of immunobiotic *L. plantarum* to diminish the replication of the virus is the reduction in the expression of entry receptors. In our study, the treatment of Calu-3 cells with *L. plantarum* MPL16 or CRL1506 did not induce modifications in the expression levels of *ACE2* or *TMPRSS2* before (data not shown) or after (Figure 4) the challenge with SARS-CoV-2 in comparison with control cells. In support of our findings, comparative studies performed on Calu-3 and Caco-2 cells and evaluating the profile of entry receptors for SARS-CoV-2 found that the expression of *ACE2* and *TMPRSS2* in Calu-3 cells were 1 to 4-fold and 4 to 64-fold higher, respectively, than in Caco-2 cells [35]. Interestingly, the work also found that the infectivity of SARS-CoV-2 was similarly efficient in both cell lines. These results indicate that considerable large variations in the expression of the receptors would be necessary to achieve inhibition of viral replication by this mechanism, and therefore, the modulation of entry receptors expression would not be involved in the protective effect against SARS-CoV-2 induced by immunobiotic lactobacilli.

Upon infection, SARS-CoV-2 also induces the expression of hundreds of genes in respiratory epithelial cells hallmarked by a significant up-regulation of cytokines and antiviral factors [34]. Transcriptional studies in Calu-3 cells found that the coronavirus increased the expression of *TLR3* and the canonical RIG-I-like receptor for RNA virus recognition *DDX58*. Furthermore, a gradual up-regulation of *IRF7, IRF9, STAT1* and *STAT2* genes was observed in Calu-3 cells after the infection with SARS-CoV-2 for 24 h [34]. In line with these findings, it was demonstrated that challenging Calu-3 cells with SARS-CoV-2 was able to stimulate the JAK/STAT signaling pathway that drives the production of type I (IFN-α/β) and type III (IFN-λ) IFNs, which constitute an important arm of the mammalian innate antiviral immune response [6]. Of note, using Calu-3 cells it was reported that SARS-CoV-2 viral replication is inhibited by type I IFNs treatment in a dose-dependent manner [6]. Furthermore, it was demonstrated that the inhibition of the antiviral JAK/STAT signaling pathway in respiratory epithelial cells significantly enhances SARS-CoV-2 multiplication [6]. Comparative studies aimed to evaluate type I IFNs sensitivity of SARS-CoV-2 relative to SARS-CoV reported that while SARS-CoV-2 maintains similar viral replication to SARS-CoV in Calu-3 cells, the novel coronavirus is much more sensitive to type I IFNs [40]. Then, the early production (or administration) of type I IFNs efficiently counteracts SARS-CoV-2 replication in respiratory epithelial cells [41]. These findings indicate that immunobiotic *L. plantarum* strains like MPL16 and CRL1506 would be able to improve the resistance to SARS-CoV-2 infection through the increase in the production of IFN-β and the subsequently enhanced expression of the viral-sensing receptors *TLR3* and *DDX58* as well as the antiviral factors *Mx1* and *OAS1* that help limit viral replication. Accordingly, the treatment of Calu-3 cells with non-viable lactobacilli was not able to modulate IFN-β production or reduce virus titers as discussed below.

Our results also suggest that the change in the cytokine profile induced by immunobiotic *L. plantarum* MPL16 and CRL1506 would not only help to limit viral replication but could also collaborate in reducing the inflammatory damage caused by the dysregulated immune response. It was shown that delayed IFN response is associated with the induction of a strong inflammatory response, which results in immunopathology and severe COVID-19 [42]. In addition, SARS-CoV-2 infection is able to strongly activate the nuclear factor kappa B (NF-kB) signaling pathway in the respiratory epithelium leading to the upregulation of inflammatory cytokines/chemokines [34]. Transcriptomic studies revealed remarkably enhanced expressions of CCL2, CXCL8, CSF3, CSF2, and CXCL10 in Calu-3 cells infected with SARS-CoV-2 [34], inflammatory factors that were shown to be strongly elevated in the serum of patients with severe clinical symptoms of COVID-19 [43,44]. In line with these previous studies, we observed here that SARS-CoV-2 infection increased CXCL8, CCL5, and CXCL10 production in Calu-3 cells. Of note, *L. plantarum* MPL16 and CRL1506 significantly reduced these chemoattractants for neutrophils and T cells. Taking into consideration that epithelial cells have the ability to influence the recruitment and activation of immune cells in the respiratory tract [45], it is tempting to speculate that the improvement of IFN-β and the reduction in CXCL8, CCL5, and CXCL10 induced by immunobiotic *L. plantarum* would contribute in vivo to control inflammatory damage. Furthermore, we have previously demonstrated that the nasal priming of mice with *L. plantarum* CRL1506 increased lung CD3^+^CD4^+^IL-10^+^ T cells after poly(I:C) administration or RSV infection, contributing to the control of inflammatory-mediated lung tissue damage [17]. Thus, the evaluation of the potential anti-inflammatory protective effects of the MPL16 and CRL1506 strains in the context of SARS-CoV-2 infection, through the modulation of the respiratory epithelium and/or regulatory T cells responses, warrants further investigation.

Our previous works demonstrated the strain-dependent ability of lactobacilli to beneficially modulate in vivo the antiviral immune response in the intestinal [24,46] and respiratory [17,18,19] tracts. In addition, our transcriptional studies performed in intestinal epithelial cells cultures revealed the strain-dependent capacity of lactobacilli to modulate the TLR3-mediated antiviral immune response and the resistance to rotavirus infection [23,24,47]. The results obtained here in respiratory epithelial cell cultures are in line with those previous findings. While *L. plantarum* MPL16 and CRL1506 differentially modulated the response of respiratory epithelial cells triggered by TLR3 activation and increased the resistance to SARS-CoV-2 infection, the strains *L. plantarum* MPL18 and CRL1905 were not capable of inducing those benefits. Furthermore, although both MPL16 and CRL1506 strains were able to beneficially modulate the antiviral immune response of Calu-3 cells, *L. plantarum* MPL16 achieved this effect more efficiently. Thus, the results presented here emphasize the need for exhaustive studies of the specific strains that could be used to protect against particular viral infections, including those produced by SARS-CoV-2.

We note with interest the fact that non-viable *L. plantarum* MPL16 or CRL1506 were not capable of modulating the poly(I:C)-triggered immune response or the resistance to SARS-CoV-2 in Calu-3 cells. This was unexpected as our previous in vivo studies showed that HK *L. plantarum* CRL1506 is able to increase the production of type I IFNs, IL-6 and the number of CD11c^+^CD11b^low^CD103^+^ dendritic cells in the respiratory tract of mice nasally challenged with the TLR3 agonist poly(I:C) or infected with RSV [17]. These contrasting results were also reported by Rosenberg and colleagues. The researchers demonstrated that the nasal priming of mice with the HK immunobiotic *L. plantarum* NCIMB 8826 provided protection against the lethal sequelae of PVM [14,15,16] and IFV [48] infections. The in vivo protective effects of the NCIMB 8826 strain were not related to the reduction in virus replication and clearance, but to efficient regulation of the inflammatory response that minimized the lung damage [15,16,48]. Of note, studies in two cell culture models, MLE-12 cells (mouse lung type II alveolar epithelial like-cells) and mTECs cells (polarized mouse tracheal epithelial cells) revealed the inability of HK *L. plantarum* NCIMB 8826 to reduce the production of CCL2, CCL5, CXCL1, and CXCL10 induced by IFV infection, using a protocol that replicated experimental conditions that were effective in vivo [48]. These, and our results presented here indicate that the ability of nasally administered HK *L. plantarum* to induce modifications in the respiratory antiviral immune response in *vivo* would depend on the interaction of lactobacilli with immune cells and not with epithelial cells of the respiratory tract. Macrophages and/or dendritic cells would be the cells modulated by HK lactobacilli as suggested by our previous studies [17,18,19]. These results indicate that the possibility of using non-viable lactobacilli to increase the resistance to SARS-CoV-2 should not be ruled out, since in vivo results could be different from those obtained in epithelial cell cultures. Therefore, the ability of HK *L. plantarum* MPL16 or CRL1506 to modulate the immune response to SARS-CoV-2 should be investigated in vivo since the use of non-viable lactobacilli is an interesting alternative to improve the immunity of immunocompromised patients in which the administration of viable microorganisms could represent a risk.

## 5. Conclusions

To date, the potential beneficial effects of immunomodulatory probiotics on SARS-CoV-2 infection and COVID-19 outcome have been extrapolated from studies carried out in the context of other viral pathogens such as IFV, RSV or PVM [7,8]. To the best of our knowledge, this is the first demonstration of the ability of immunomodulatory lactobacilli to positively influence the resistance against SARS-CoV-2 infection. The in vitro results presented in this work provide evidence that immunomodulatory *L. plantarum* differentially modulate the innate antiviral immune response in respiratory epithelial cells and favorably influence their resistance to SARS-CoV-2 infection. Further mechanistic studies and in vivo experiments in SARS-CoV-2 infection in animal models are necessary to identify specific strains of beneficial immunobiotic lactobacilli such as *L. plantarum* MPL16 or CRL1506 for the prevention or treatment of COVID-19.

## Data Availability

All the data related to this project are presented here.
